# “It comes altogether as one:” perceptions of analytical treatment interruptions and partner protections among racial, ethnic, sex and gender diverse HIV serodifferent couples in the United States

**DOI:** 10.1186/s12889-022-13528-8

**Published:** 2022-07-09

**Authors:** Danielle M. Campbell, Karine Dubé, Portia D. Cowlings, Patricia Dionicio, Rowena M. Tam, Harsh Agarwal, Jamila K. Stockman, Judith D. Auerbach, John A. Sauceda, Amy A. Conroy, Mallory O. Johnson

**Affiliations:** 1 Charles R. Drew University of Medicine and Science (CDU), 1731 East 120th Street, Los Angeles, CA 90059 USA; 2 Joint Doctoral Program in Public Health, University of California, San Diego/San Diego State University, 9500 Gilman Drive, La Jolla, CA 92093 USA; 3 Division of Infectious Diseases and Global Public Health, Department of Medicine, University of California, San Diego (UCSD), 9500 Gilman Drive, La Jolla, CA 92093 USA; 4 UNC Gillings School of Global Public Health, 4108 McGavran-Greenberg Hall, Chapel Hill, NC 27599 USA; 5 Graduate School of Education and Psychology, Department of Education, Pepperdine University, 6100 Center Drive, Los Angeles, CA 90045 USA; 6 San Francisco (UCSF) Department of Medicine, Division of Prevention Sciences, Center for AIDS Prevention Studies (CAPS), University of California, 550 16th Street, 3rd Floor, San Francisco, CA 94158 USA

**Keywords:** HIV cure research, Analytical treatment interruptions, Partner protection measures, HIV serodifferent partners, Couples, Socio-behavioral research, Sexual and gender minorities, Racial and ethnic minorities

## Abstract

**Background:**

Most HIV cure-related studies involve interrupting antiretroviral treatment to assess the efficacy of pharmacologic interventions – also known as analytical treatment interruptions (ATIs). ATIs imply the risk of passing HIV to sexual partners due to the loss of undetectable HIV status. There has been a notable lack of attention paid to perceptions of ATIs among racial, ethnic, sex and gender minorities, and HIV serodifferent couples. These populations are among those most impacted by HIV in the United States. Future HIV cure research paradigms should equitably include considerations from these groups.

**Methods:**

From August – October 2020, we conducted in-depth interviews with 10 racial, ethnic, sex, and gender minority HIV serodifferent couples in geographically diverse regions of the United States to understand their perspectives about ATIs and partner protection measures to prevent secondary HIV transmissions because of participation in ATI studies. We used framework analysis to analyze the qualitative data.

**Results:**

Of the 10 couples recruited, four identified as a gay couple, two as a gay and bisexual couple, two as a heterosexual couple, one as a gay and queer couple, and one as a queer couple. We found that HIV serodifferent couples in our study viewed ATIs as contradicting HIV treatment adherence messages. Couples expressed discomfort around ATIs in HIV cure research. They were concerned with the return of HIV detectability and worried ATIs might result in secondary HIV transmission. Participants were strongly in favor of using a range of partner protection measures during ATIs that included PrEP, HIV risk reduction counseling, and alternatives for penetrative sex practices. Couples also recommended that sex partners be consulted or involved as part of ATI trials.

**Conclusions:**

Our findings highlight new potential opportunities and strategies to mitigate risk of HIV transmission during ATIs among key groups historically under-represented in HIV cure research. Findings also underscore the relational aspects of ATI trials. We provide preliminary considerations for planning ATI trials with diverse HIV serodifferent partners. Future studies should continue to explore these issues among other types of partnerships, cultures, and socio-cultural settings.

**Supplementary Information:**

The online version contains supplementary material available at 10.1186/s12889-022-13528-8.

## Introduction

When people living with HIV adhere to antiretroviral therapy (ART) they effectively have no risk of transmitting HIV to their sex partner(s) [1]. This reality has formed the basis of a global public health campaign known as undetectable equals untransmittable (U = U). However, undetectable does not equal cured as current ART does not eliminate HIV reservoirs, which remains the main barrier to curing HIV [2]. Most HIV cure-related studies are in the early experimental phase and aim to either completely eliminate HIV from the body or achieve sustained viral suppression without the need for ART [3]. The design of most of these studies requires the interruption of ART, also known as analytical treatment interruption (ATI) [4, 5]. Besides clinical risks associated with interrupting treatment, there is the risk of secondary HIV transmission to sex partner(s) due to the loss of the undetectable HIV status, which obviates the U = U equation [6]. Two secondary HIV transmission events in the context of HIV cure trials have been documented among sex partners [7, 8], raising the ethical question of whether HIV cure studies with ATIs must include risk mitigation interventions for sex partners of trial participants [9–13].

Moreover, HIV cure studies critically lack representation of racial, ethnic, sex, and gender diverse populations, who bear the brunt of HIV diagnoses in the United States (U.S.) [14]. A 2019 landscape analysis of HIV cure clinical research revealed that participants in HIV cure trials are not generally representative of populations most adversely affected by HIV [15, 16]. With few exceptions, there is a dearth of research on how groups historically absent or excluded from HIV cure research perceive participation in these types of studies in general and about ATIs specifically [16]. One study of Black/African American transgender women’s opinions found strong aversion towards interrupting ART [17]; and a focus group study with mostly Black/African American people with HIV (PWH) in the Northwestern U.S. found satisfaction with current ART and concerns regarding interrupting life-saving treatment [18].

In addition to the lack of racial, ethnic, sex and gender diversity among HIV cure research participants, there is a notable lack of attention to perceptions of ATIs among HIV serodifferent couples – in which one partner has HIV and the other partner does not. HIV transmission risk among serodifferent couples when the partner with HIV is suboptimally adherent to ART is estimated to be amongst the highest of all transmission categories [19]. Given this risk, understanding the perspectives of HIV serodifferent partners is important for many reasons: 1) risk of secondary HIV transmission increases when the partner with HIV is taken off ART [20], 2) HIV prevention and treatment decisions are often made as a couple [21], 3) primary partners influence one another’s behaviors [22], 4) partner considerations, if not taken into account could limit participation in ATI research, and 5) perspectives of HIV serodifferent partners can inform protocols to protect partners during ATIs. In the context of ATI research [23], there is a need to gather perspectives from diverse PWH [16].

We conducted a qualitative study among HIV serodifferent couples in the U.S. who were diverse by race, ethnicity, sex, gender and geographic region to: 1) understand experiences with current HIV medications and perceived improvements in ART regimens as these affect perceptions about ATIs, 2) explore how HIV serodifferent partners perceive ATIs, 3) assess acceptable partner protection measures to prevent unintended HIV transmission events during ATIs, and 4) generate considerations for partner involvement in HIV cure trials involving ATIs.

## Methods

### Sample

We used a purposive, non-probabilistic sampling technique to recruit HIV serodifferent couples in the U.S. Couples were defined broadly as two individuals considered to be in a relationship – either romantically or sexually. One of the lead authors (D.M.C.) led recruitment activities and advertised the study to various community-based organizations (CBOs) working on HIV in the U.S. using an institutional review board (IRB)-approved recruitment flyer. We focused on CBOs whose mission was to explicitly serve racial, ethnic, sex, and gender minority individuals. Study candidates contacted D.M.C. by phone and asked for details about the study. For couples to be eligible, both partners had to be at least 18 years of age and report being in an HIV serodifferent relationship. In addition, participants had to self-identify as being from a racial, ethnic, sex, and/or gender minority group and be willing to be interviewed as a pair. As a safeguard, only couples in which *both* partners had disclosed their HIV status to each other were considered. Further, the importance of maintaining confidentiality was emphasized during and after the interviews.

Upon confirming interest and eligibility, D.M.C. and a research assistant (P.D.C.) emailed the relevant study documents, including the IRB-approved informed consent form, demographic sheet, and study fact sheet. Upon confirming the day and time of each interview with both partners, the research team (D.M.C. and P.D.C.) sent Health Insurance Portability and Accountability Act (HIPAA)-compliant virtual conferencing weblink to each informant. Couples were interviewed remotely as a pair, and couple identification numbers were assigned sequentially on the day of each interview.

We used in-depth interviews to elicit rich and candid dialogues around ATIs and partner protection measures. Interviews involving both partners together allowed participants to co-construct meaning and stimulate ideas around a topic that involved two sides [24, 25]. Interviews also fostered an atmosphere of safety and openness around a sensitive topic: sexual practices and partner protections in HIV serodifferent relationships [24].

### Data collection

The interviewer (D.M.C.) conducted all interviews in English using a virtual conferencing platform with the IRB-approved interview guide (Table 1). The study team was diverse along the lines of race, ethnicity, gender, and sexual orientation. Although no member of the study team was part of an HIV serodifferent relationship, one of the lead authors has expertise in conducting research among HIV serodifferent couples. Additionally, investigators have many years of experience conducting community-engaged research on HIV prevention, treatment, and cure.

**Table 1:** In-depth interview guide – perceptions of ATIs and partner protections among racial, ethnic, sex and gender diverse HIV serodifferent partners in the United States (United States, 2020 – 2021)

**Introduction** • First, thank you so much for your time in completing today’s couples’ interview. • Are you both in a safe and comfortable place where you both are able to actively participate for the full duration of today’s discussion? **Interview Questions** • Can you please describe your experiences with current HIV medications? • Have you heard of the expression U = U (Undetectable = Untransmittable)? • What are your thoughts about analytical treatment interruptions used in HIV cure-related studies? • How does your understanding of U = U fit within the context of an HIV cure-related study with an ATI? • Do you think safety provisions should be put in place to protect partners throughout your participation on an HIV cure-related study? • Do you think it would be beneficial to offer pre-exposure prophylaxis (PrEP) to sexual partners of participants enrolled in HIV cure-related research trials during treatment interruption phase of the study? • Should anything else besides PrEP be offered to either the study participants or their partners? If so, what should be offered? • Would you consult your partner if you had to make a decision about whether to participate in an HIV cure-related research?	

Interview questions were informed by constructs of the theory of planned behavior [26] and prior literature on HIV cure research, including a recent article summarizing research priorities with regards to socio-behavioral sciences and ATIs [27]. Interviews lasted between 45 and 90 minutes. After each interview, the interviewer (D.M.C.) took detailed field notes about key observations. A research assistant (P.D.C.) updated study management tools, such as demographic logs. Each partner received an electronic payment of $25 following each interview.

### Data analysis

All interviews were professionally transcribed, using color codes to differentiate between the interviewer (D.M.C), the partner with HIV, and the partner without HIV. A research assistant (P.D.C.) reviewed all transcripts for accuracy and completeness against the audio recordings.

We used framework analysis to analyze the qualitative data [28]. Framework analysis employs matrices to organize data into emergent themes and subthemes, allowing researchers to compare findings between respondents, while maintaining links to exemplary quotes. After carefully reading each transcript, a primary coder (K.D.) created a summary table with columns for each question of the interview guide, and rows for each study participant, color-coded by HIV-status of the respondent. A secondary coder (H.A.) reviewed the data matrix and added any important observations that were missed by the first coder. Data were analyzed on both individual and couple levels [25]. We also highlighted text units where exchanges between the partner with HIV and the partner without HIV were distinctly generative. Key themes and sub-themes were summarized into the framework analysis table (in Microsoft Excel), highlighting areas of convergence or divergence between the partners and the pairs (although most pairs converged on most themes). Due to the relatively small sample size (10 couples) and the diversity of experiences in the lives of HIV serodifferent couples [29], we may not have reached saturation [29]. Finally, we wrote narratives to summarize the data and resolved discrepancies by consensus.

### Ethics statement

The Charles R. Drew University of Medicine and Science (CDU) and the University of North Carolina at Chapel Hill (UNC-CH) IRBs approved the study with written informed consent. All methods were carried out in accordance with relevant guidelines and regulations, including the Declaration of Helsinki.

## Results

### Study participants

In total, we recruited 20 individuals in HIV serodifferent partnerships throughout the U.S. between August and October 2020 (Table 2). Of the 10 couples, four identified as a gay couple, two as a gay and bisexual couple, two as a heterosexual couple, one as a gay and queer couple, and one as a queer couple. With respect to gender, 15 individuals identified as cisgender men, 2 as cisgender women, 1 as a transgender woman, and 2 as gender non-conforming/non-binary individuals. Participants self-identified as Black/African American (*n* = 7), Latinx/Chicanx/Hispanic (*n* = 6), and White/Caucasian (*n* = 7). Participant’s age ranged from 36 to 73 years. Although not an inclusion criterion, all participants living with HIV were taking ART at the time of the interview. There were 3 couples from Los Angeles, CA; 3 from San Francisco, CA; 2 from Baltimore, MD; 1 from New York, NY and 1 from Durham, NC (not included in Table 2 to protect anonymity).

**Table 2:** Characteristics of HIV serodifferent partners (*n* = 10 couples; *n* = 20 participants)

ID	Participant Characteristics	Comments
01	**Partner with HIV**: Cisgender female, 51 years old, Black/African American, diagnosed 1991, on ART **Partner without HIV**: Cisgender male, 51 years old, Black/African American	Heterosexual couple
02	**Partner with HIV**: Cisgender male, 49 years old, Latino, diagnosed 2003, on ART **Partner without HIV**: Cisgender male, 59 years old, Latino	Gay/same gender-loving couple
03	**Partner with HIV**: Cisgender male, 36 years old, Black/African American, diagnosed 2010, on ART **Partner without HIV**: Cisgender male, 36 years old, Black/African American	Gay/same gender-loving couple
04	**Partner with HIV**: Cisgender female, 65 years old, Black/African American, diagnosed 1998, on ART **Partner without HIV**: Cisgender male, 66 years old, Black/African American	Heterosexual couple
05	**Partner with HIV**: Transgender female, 50 years old, Chicana, diagnosed 1995, on ART **Partner without HIV**: Cisgender male, 62 years old, White/Caucasian	Gay/same gender-loving and bisexual couple
06	**Partner with HIV**: Cisgender male, 43 years old, White/Caucasian, diagnosed 2008, on ART **Partner without HIV**: Cisgender male, 37 years old, Black/African American	Gay/same gender-loving and queer couple
07	**Partner with HIV**: Cisgender male, 54 years old, Latino, diagnosed 1998, on ART **Partner without HIV**: Cisgender male, 49 years old, Latino	Gay/same gender-loving couple
08	**Partner with HIV**: Gender non-conforming male, 42 years old, White/Caucasian, diagnosed 2009, on ART **Partner without HIV**: Cisgender male, 37 years old, Latino	Queer couple
09	**Partner with HIV**: Non-binary, 73 years old, White/Caucasian, diagnosed 1983, on ART **Partner without HIV**: Cisgender male, 71 years old, White/Caucasian	Gay/same gender-loving and bisexual couple
10	**Partner with HIV**: Cisgender male, 62 years old, White/Caucasian, diagnosed 1983, on ART **Partner without HIV**: Cisgender male, 38 years old, White/Caucasian	Gay/same gender-loving couple

### Experiences with current HIV medications and perceived improvements in ART regimens

To assess overall satisfaction with HIV treatment and willingness to interrupt ART to participate in an HIV cure study, we asked participants living with HIV to describe their positive and negative experiences with ART. All participants with HIV reported being very satisfied with their current HIV medication and expressed contentment with one-pill-a-day regimens. Participants reported experiencing few side effects and good tolerability of their current HIV treatment regimen.

I don’t think bad about them [HIV medications]. I’m glad that it’s broken down to one pill instead of taking multiple pills. So it’s not a bad thing. It’s like I’m programmed on taking them. And I also take medication for mental health, depression. – 03-Partner with HIV

Most participants with HIV described how they would be hesitant to switch to an entirely new antiretroviral regimen. One reason given was because some of the newer medications (e.g., Dolutegravir) were associated with physical side effects, such as weight gain.

Participants without HIV noted that current HIV medications kept their partners healthy and expressed hesitancy around having their partners changing their HIV medication.

What happens in this instance is that [the partner with HIV] presently is doing a wonderful life, and she’s not having any problems with the regimen of medicine that she’s on, and I just don’t see any sense in tampering with that, you know. I mean, what more could you promise her? She’s undetectable. I mean, if she’s not going to take a pill then that means that whatever she’s been taking has cured her and there’s no need for a pill… Other than that, hell no. – 01-Partner without HIV

We also asked participants to give their perspectives on new, long-acting ART regimens (LA-ART) becoming available in oral and injectable formulations as these could affect thinking about ATIs. Both participants with HIV and those without expressed mixed feelings about LA-ART, although four participants with HIV expressed willingness to try it. The perceived benefits were an improvement over daily oral medication and a step towards getting closer to an HIV cure, and alleviating concerns around having to remember to take medications. Concerns around having to remember to take daily medication emerged for both participants with and without HIV.

Yeah. You know, I never talk about it, but I think that would change the whole thing. Because right now, I know that I have to take HIV meds – otherwise, I will get sick because it already happened. I will get less anxiety, I will have less medication through my body, through my system. That will change a lot. For me especially, because you know by taking medication, I have to be at work, but I have to take a lunch. If I forget or don’t take a break, the viral load’s going to get higher, so I have to be aware about all these things. And by finding the cure, by taking whatever medication will be the cure, that will be something really good, at least for me. – 02-Partner with HIV

It would be better if they don't have to worry about taking the medicine—they forget about it sometimes or worry about it. That's what I think. – 02-Partner without HIV

Perceived barriers to LA-ART included concerns about injections:

I don’t like injections and then I bruise easy. So that would be like a problem for me, because then I’d look like I was a intravenous user. – 01-Partner with HIV

Both participants with HIV and those without expressed wanting to receive more information about emerging treatment options, such as LA-ART.

### Understanding of Undetectable = Untransmittable (U = U)

We asked HIV serodifferent couples to describe their thoughts about the U = U public health message, as this may also affect perceptions of ATIs. We did not initially provide the meaning of U = U during the interview, but later explained it to participants if they were unfamiliar with the expression. Participants had a mixed understanding of U = U. One couple specifically reported relying on U = U as their primary protection method of preventing HIV. Approximately one-third of participants had previously heard of U = U and were able to explain its meaning.

So not only for myself but being in a relationship that they’re taking into account in the U = U era that his health is going to be okay and that I’m not going to transmit HIV to him while I’m in that interruption. Anything that looks outside of that, I’m going to have a hard time trusting. – 06-Partner with HIV

My understanding of U = U is just that, that once you reach the point where you’re undetectable, that you are no longer transmissible. So you are no longer at-risk for transmitting HIV, no matter what your status. That’s pretty much the extent of it, but I think it’s pretty clear. – 08-Partner without HIV

Participants engaged in dialogue around the meaning of U = U for them and other HIV serodifferent partners, as evidenced by the following exchange.

Yeah, U = U for me is undetectable is untransmittable. Like for myself, if I control the virus in my blood, I will not pass it over to my partner. – 02-Partner with HIV

…Yeah, what I notice is when people are undetected, they don’t have the virus in their blood, so it’s okay if people have sex. Of course, [we are] taking precautions. – 02-Partner without HIV

Three participants interpreted U = U as being different from the Undetectable = Untransmittable message. They ascribed different meanings to U = U, such as keeping each other informed and equality in the relationship when caring for one another.

Treat me the way you would want to be treated. I mean, just keep me informed. Not a whole lot. Just keep me informed as to what is going on and the decision is mine if I want to have intimacy.

– 01-Partner without HIV

Equality – 01-Partner without HIV

Does that mean like you care about me and I care about you? I think that's the way I got it.

– 07-Partner with HIV

Two participants with HIV had not previously heard of U = U and learned about its meaning during the interview.

I’m not detected. That’s a good thing… No, I never heard that. – 03-Partner with HIV

That's a cute word. I'm virally suppressed… I’m virally suppressed! – 04-Partner with HIV

One couple showed divergence in understanding what U = U meant. The partner with HIV explained the meaning of U = U to their partner during the interview, as shown by the following exchange.

Well, I can answer that, but I want [name of partner] to answer it first; see if he actually knows the phrase… – 09-Partner with HIV

… I don't. Sorry, I don't know what it means… – 09-Partner without HIV

… Undetectable. Well, if my viral load is undetectable, then I can't transmit the virus in any of my bodily fluids including my blood. So I would be protecting anyone that I was being sexual with, and that includes not being safe. – 09-Partner with HIV

### Perceptions of Analytical Treatment Interruptions (ATIs)

Most participants – both with and without HIV – expressed strong worries and discomfort associated with the thought of interrupting ART to advance HIV cure science. Some viewed contradictions between ATIs and deeply entrenched messages around the need for ART adherence to keep HIV suppressed to stay healthy.

For me, I feel like it could be danger… I've been learning and I know how if you stop taking your medicine that the T cells will replicate. I don't know the terms, the medical terms, but… I don't know exactly what is the danger and what is not, because I've been taught to take my medicine every day. So I never—and I'm very responsible person at any level. So if you tell me to take my pills at 10, you know, one minute or two before, I have my pill in my hand, every day. – 07-Partner with HIV

I really admire [partner]'s religiously taking his medications and taking care of himself for so many years. It's kept him alive and healthy, and I would be very, very concerned if he did anything that would potentially compromise his health. – 09-Partner without HIV

Most participants expressed worries about the possible negative side effects they may experience with interrupting ART including: an increase in viral load, decrease in T cells, resistance to HIV medications, and development of opportunistic infections. Two participants with HIV recounted how they previously interrupted ART and witnessed changes in their body almost immediately, which caused substantial stress and worry.

Now I was just thinking about if I miss a couple of days of my medications I would start to—some bumps start coming on my feet... I’ve had some days that, like, some time ago when I would miss a couple of days I would have a reaction. So I knew that I had missed a couple of days. But that would be another one, yeah. Becoming resistant… Yeah. Like did I mess it up? Am I going to have to be switched to another medication? … Have I triggered HIV to – Oh, my God. What do they call it? Getting an opportunistic infection. – 01-Partner with HIV

I've already tried to stop taking my medication and I got sick… I think for me and for some people, my main concern would be, is that medication going to work on my body the way it should be? You know, like control or kill the virus in my body? So that's why I think my concern would be, is that going to work in my body the way it should be or am I going to get sick? That's what I'm thinking. Like I told you, I did it before, not to take my meds because I got like, “Okay, I'm feeling good, I control the virus, I'm sleeping good, I'm eating good, I'm resting.” But then the virus was in my body and I got sick. – 02-Partner with HIV

Three participants with HIV associated ATIs with the possibility of clinical complications or even death.

What about if the thing goes so wrong that somebody can die out of your research, because they stop taking the medicine? – 07-Partner with HIV

Most participants without HIV expressed discomfort around allowing their partners to be off HIV medications. One participant without HIV disapproved of ATIs entirely:

But what would it matter in a [situation] in particular where you’re HIV undetected, and if it ain’t broke why tamper with it? Why fix it if you’re HIV undetectable and you can’t pass the HIV virus, what’s the need for a new therapy is my question? … If I’m HIV undetectable and if I cannot pass it on to anyone whether it’s my husband or whoever, I mean, what’s the purpose of messing with that? … I mean, that’s ludicrous, I think. But I guess in these type of research settings these questions has to be asked, but I would never, ever ask that question if it’s fixed. If it’s fixed leave it alone. – 01-Partner without HIV

Participants viewed ATIs as disrupting their ability to use U = U as a protective measure to prevent HIV transmission. One participant with HIV described how he would want to feel empowered to communicate the loss of undetectable HIV status during an ATI.

Of course the concern would be there… You know, I didn't have a confidence in being undetectable anymore. And so, certainly, ideally if this were something that I would be considering, then I would really prefer to know that I'm getting viral loads checked on at least a monthly basis, so I would have more of a sense of what's really going on in my body, day to day. Because that would help me… how much I feel I need to disclose with other people. And certainly, in my immediate circle, I would probably have a conversation at the start of this saying, "I'm going to be in this study, and we're going to have to see how this goes," just so there was kind of a generalized awareness that this is something going on with me… And if there's anything I need to communicate, then I feel empowered to communicate it. – 08-Partner with HIV

One couple carefully considered the implications of no longer being undetectable for HIV. These included the need for frequent viral load measurements and robust partner protection measures until the restoration of HIV undetectability, and concerns about HIV-related criminalization:

Well, I think at that point it becomes a matter of measurement. I mean, if you are interrupting treatment then U = U would only apply if you were still undetectable. And so, it would be a matter of having enough frequency of testing to really monitor and be able to say with any kind of certainty that I am undetectable whenever I'm also not—or when I'm in between check-ins. Because if you can't guarantee that you are undetectable—if there are spikes and valleys, if there are shifts there… I think you would start having conversations around condom use and around other preventive measures again, until you can verify that you are no longer detectable… – 08-Partner without HIV

… it can be a matter of criminality to be HIV positive and not share your status, particularly if you are infectious. So, obviously, I would not want to find myself in a situation where I was legally liable for putting someone else at an actual risk. – 08-Partner with HIV

Only one participant with HIV described ATIs as scientifically necessary to see the full effect of experimental interventions towards an HIV cure.

### Worries around transmitting and acquiring HIV

All participants with HIV expressed concern about transmitting HIV to their partners during ATIs. In turn, participants without HIV expressed concern about acquiring HIV if their partner(s) became detectable and would want to know the viral load test results of their partner(s) at regular intervals. Participants also worried about whether experimental interventions would keep HIV suppressed, what would happen if condoms broke during sex, and how their sex life might be affected by ATIs. The following exchange with one couple is illustrative:

Oh, would I be [concerned]—oh, of course. I definitely would. – 03-Partner with HIV

…Okay, well, I would be concerned because it’s like a[n] experiment as far as I know that they’re providing you with a certain type of medicine that’s supposed to work like the other medicine. Is that the case? … Because you said it was a drug associated with it. Okay, yeah, so I know that we use condoms but I would be concerned with him being detectable again… Yeah. I would worry about him being detectable because I wouldn’t want him to, you know, if anything with like the condom breaking or anything of that nature happening, like where it’s though it’s a chance that I may be affected… – 03-Partner without HIV

…I also, with as much as the love that we have for one another, as much I truly—I know he genuinely cares about me and love me, I wouldn’t want that to change, because probably in thought I was saying it wouldn’t change anything. I know if it was to actually happen we never know what we think until it actually happens. We all can say what we would do, but until it actually faces that and, you know, I wouldn’t want it to change anything. Do you know what I mean? Because he is negative and I want him to stay that way. – 03-Partner with HIV

…And also not even that. Like if we were going to do that or take that chance to do that, I think that it might [inaudible] our love life or affect us, our sexual love life in a way. – 03-Partner without HIV

…Which I think we would want to take precautions. He don’t want to be at risk, so we probably wouldn’t do it [have sex] nowhere near as much. – 03-Partner with HIV

Two couples also expressed fear that transmitting HIV could negatively affect the love between the partners.

Yeah, because I don't want him to get sick, you know? Because he's negative and I just...the love will be gone. – 05-Partner with HIV

Others were worried about transmitting HIV to secondary sex partners.

We don't exist in, like, a monogamous bubble, and so that shift would affect the people who we engage with… and sexually engage with. – 06-Partner without HIV

Well, we do play with others, so I think that is a significant thing to lift up, 'cause sometimes I worry that with research that people sort of fall into really just focusing on the primary partner. – 06-Partner with HIV

### Partner protection measures

All couples were strongly in favor of robust partner protection measures during ATIs. There were important variations between the couples around the acceptability of pre-exposure prophylaxis (PrEP), condoms, and other partner protection measures. Partner protection measures would also vary depending on whether partners were in a monogamous versus non-monogamous partnership.

One participant with HIV described how ATIs would require an extension of the couple’s current practices to prevent HIV transmission during sex.

I think, you know, like right now even though we are not participating or in any clinical research studies, we are taking precautions so he doesn't get the virus. So I think if we are participating in one of these clinical research studies, even though we are taking the medication that's supposedly going to get the cure, I think even at that moment we have to take care of each other, especially me to take care of him. – 01-Partner with HIV

Approximately half of the participants were strongly in favor of offering PrEP to the partner without HIV during an ATI.

They should be on PrEP. – 10-Partner with HIV

…Oh yeah, like if you offered them PrEP. For me I get it easily, so it's not something I think about. But I guess people in other situation, they wouldn't have PrEP readily available to them. – 10-Partner without HIV

…And it should be made available to them. – 10-Partner with HIV

However, the second half of the participants raised concerns around PrEP, including lack of trust in the prophylaxis, possibility of side effects, and costs. Participants also insisted on removing financial barriers to PrEP access for partners without HIV during the ATI.

You can take it but I'm not—I'm gonna give you no hanky panky, uh-uh… I don't trust that PrEP… I heard the PrEP damages your liver? – 05-Partner with HIV

I didn’t really feel like it was necessary for me to take it because I have heard that Truvada or PrEP had side effects with different people. And by me already having high blood pressure, I just don’t feel like it’s necessary to put any type of medicine in me if I really don’t need it. – 03-Partner without HIV

Yes. Absolutely… Because it's expensive as hell. And it shouldn't be challenging—you know, people should have access to it. It's life-saving medication and if you're going to participate in this, then you should try to remove as many financial barriers that might discourage people. – 06-Partner without HIV

Three couples recommended the use of condoms during an ATI. In addition, two older couples reported never having sex without condoms.

[W]e never had sex without a condom… – 10-Partner without HIV

…Yeah. – 10-Partner with HIV

...for all those years, and I never got HIV, so... – 10-Partner without HIV

In contrast, a younger couple reported that condomless sex had become the norm in the community, given U = U, and described how they would not be responsive to messages focused on condoms or dental dams.

I mean, in our relationship, we don't use condoms, we're not going to be using condoms. It's not going to happen. So we're going to need to make sure that he's absolutely stable on his PrEP… I mean, it would be a huge removal of a barrier, so to speak, if we knew that he was going to have full access to whatever prevention items he needed. He's pretty stable and good to go on PrEP, but if there were any challenges or any potential challenges, I would want that addressed and handled up front, and I think that would make a difference. We're not going to be very responsive to condoms and dental dams. – 06-Partner with HIV

The same couple recommended making the full HIV prevention toolkit available to partners who engage in HIV cure research with ATIs and highlighted the element of choice.

So for me, that's access to the full prevention toolbox and not just some part of it or what's most convenient, so for me, it's a great opportunity to sit down and make sure that PrEP is available, both daily and on demand, making sure that post-exposure prophylaxis is readily available and that there's a clear way for a partner to access it in case that's the choice of where they want to go. That would be a non-negotiable for me—otherwise, there's no way that I'm going to sign up for that. – 06-Partner with HIV

Some couples mentioned temporary abstinence, or even celibacy as possible effective partner protection measures during ATIs. However, these measures may be more difficult to sustain during longer ATIs.

You don't want to have no relations, huh? – 04-Partner with HIV

…I don't know about all that. – 04-Partner without HIV

…You know, special relations… Yes, when I say no more relations, he wouldn't want to have no relations with somebody that's off they meds… Because he might get infected. – 04-Partner with HIV

Well, for me, I just wouldn't be sexual, but I'd have to know the result of that test [HIV viral load test]… Yes. – 09-Partner with HIV

Yeah, I agree with [name of partner]. – 09-Partner without HIV

[S]o if you have somebody else on PrEP, for example, or if you have a prolonged period of celibacy where you're not having sexual contact, just to reduce risk, I feel like that would be important. So one way or another, making sure that because of the uncertainty of what might happen without medication, you would at least have the safety of making sure that you wouldn't be a danger to anybody else during that time. – 08-Partner without HIV

To prevent HIV transmission during ATIs, some participants recommended alternatives to penetrative vaginal or anal sex, such as oral sex, mutual masturbation, pornography, or sero-positioning.

Yeah. Well, you know, at this moment we have sex is like oral, masturbation, watching porno. That kind of stuff, like no penetration. You know, that's the way we take care of each other. – 01-Partner with HIV

We do a good job. – 01-Partner without HIV

Other type of sexual behavior that doesn't involve anal intercourse I would encourage, if someone's serodiscordant and not zero viral load… Like masturbation or even oral sex, because frankly, I've never seen anybody catch AIDS from giving head, you know? I haven't. And I've talked to a lot of friends on their dying beds and talked to them about this, and it's always been an agreement—because if it were spread that easily, half the world would have been HIV positive by now. – 10-Partner with HIV

Participants insisted on the need for good communication about their health as an important partner protection strategy.

No, I mean if my partner was off medication for a couple of days, say, “Baby, look, I haven’t had my meds in a couple of days.” You know, information, let the buyer be aware… Let me make the decision whether I want to go forward. –02-Partner without HIV

[We] both communicate very well. – 03-Partner without HIV

…Yeah. So we have that. Communication is key, you know. And sometimes we’ve had thoughts about bringing in a third party but we keep vigilant but so far we haven’t and we just keep each other straight up and honest with each other. And so it won’t be no surprises at the end. – 03-Partner with HIV

Participants in openly non-monogamous relationships noted the importance of having a plan for HIV or ATI disclosure for both primary and secondary sex partners. In the context of ATIs, they proposed taking themselves out of the pool where people can play freely with others because U = U would be breached (e.g., the partner with HIV would no longer be virally suppressed).

It would be very challenging to have to go back to having more complicated plans for disclosure in those [non-monogamous] scenarios… And part of that for me is if I'm receiving the same constant [heteronormative] prevention messaging when I've indicated that certain things just don't work for me, but, like, it has to be brought up every time, for me it's very off-putting. I don't know if it's that way for other people, but I like to cover things once and then not have to keep doing it every single time. – 06-Partner with HIV

We are not in a monogamous relationship, so obviously the ways that we would mitigate risk as a couple are not necessarily the same ways that we would—that I or we would try to mitigate risk with other partners. For the past handful of years, it's sort of really felt like a moot point, to an extent, to even really discuss HIV status anymore when engaging in sexual conversations. Because for me, I have a fairly ironclad confidence that I'm staying undetectable, and undetectable means I can't transmit the virus. And add to that the fact that PrEP has been fairly overwhelmingly ubiquitous in our sexual communities, so no one uses condoms anymore, and no one discusses the use of condoms anymore, in general, unless for whatever reason they feel very strongly about bringing the topic up. So that having been said, it would definitely be a change of practice, at least temporarily, for me to have to actually ask sexual partners, "Are you taking PrEP? Here's my situation. I am positive, and I am not on my regimen right now." It would require definitely an added layer of conversation around sexual health, that certainly in the moment might, I guess, derail the energy, as it were. (Laughs.) – 08-Partner with HIV

In addition, couples noted needing adequate counseling, social support, and information about how other couples were successfully managing and re-orienting their lives around ATIs.

### Partner consultation and involvement in ATI trials

Couples provided a range of answers when asked if the partner(s) without HIV should be involved as part of ATI trials. Some couples wanted the decision to participate to be made together while others wanted both partners to be actively involved in the entire ATI clinical study.

Most participants with HIV discussed the importance of consulting with their partners about whether to participate in ATI trials.

And then my partner, of course, because there’s the increase of viral load that could put him at risk. So I would need him to know… You know, any precautions that he needs to take. – 01-Partner with HIV

Couples did not always agree about whether the partner without HIV should be involved in the decision of the partner with HIV to participate in trials with ATI.

And not so much my partner, because I've participated in things in the past without asking his permission... – 09-Partner with HIV

…[F]or something like this that could potentially negatively affect [name of partner with HIV] health, I would think that he would want to talk to me about it first and also get my input and advice. – 09-Partner without HIV

Absolutely. I mean, we're married, and we've been together 13 years. So whatever I do, it includes [name of partner]. – 10-Partner with HIV

…I feel it's on him. It's his decision. I would definitely stay on PrEP, if he did do that. But I don't think it would change our relationship in any way. I'd be more worried about him, but I would figure it's his choice. It's his body… But I think going to the meetings with him would be nice. Maybe updating me on... updating on how the research is going… Not waiting until it's over. Yeah. – 10-Partner without HIV

For some, communication was essential.

I think it's a reflection of what our standards for a relationship already are, which is open communication about everything. It's less about permission and more just about the idea of letting me know what could be happening and getting my input around that before the decision is made. – 08-Partner without HIV

Participants without HIV wanted to be actively involved in the ATI trials to provide support and attend study visits, even if they would not officially be considered participants in HIV cure trials. As one participant said:

You take one person, you have to take the second one, take the partner. Like a marriage, it comes altogether as one. – 05-Partner without HIV

## Discussion

The findings from this qualitative study advance prior socio-behavioral research on HIV cure by highlighting the varied perceptions of ATIs and partner protection measures among diverse HIV serodifferent couples in the U.S., who have been underrepresented in HIV cure research to date [10, 11]. We found that HIV serodifferent couples were highly satisfied with current ART regimens and expressed discomfort around ATIs used in HIV cure research. Participants were primarily concerned that ATIs would represent a breach of U = U principles and might result in passing or acquiring HIV. Both partners with and without HIV were strongly in favor of using a range of partner protection measures during ATIs and recommended that partners be consulted or involved as part of ATI trials. These findings highlight new opportunities to mitigate HIV transmission during ATIs and underscore the importance of engaging partners in decisions to enroll in HIV cure trials [12].

HIV serodifferent couples in our study viewed ATIs as contradicting ART adherence messages and associated them with danger. Worries around interrupting ART likewise have been observed in previous HIV cure socio-behavioral studies [16–18] – most notably among Black/African American transgender women [17]. In our study, ATIs were further perceived as a breach of the U = U message. This fundamental tension between U = U and ATIs has been documented elsewhere [30, 31], and implies that the scientific rationale for ATIs must be carefully communicated to PWH and communities in lay terms.

HIV serodifferent partners also flagged the risk of transmitting HIV to secondary partners. There is a lack of consensus on how to intervene with participants who have multiple sex partners in the context of ATIs, which a significant gap in the field. As our study participants noted, ATIs would warrant frequent viral load monitoring [4], support to disclose detectable viral load to partners, and a range of options to protect sex partners from HIV [12, 13].

Participants conveyed strong support for robust partner protection measures, including provision and/or referral for PrEP, during ATIs among HIV serodifferent partners. These findings corroborate a recent consensus statement [4] and align with World Health Organization (WHO) guidance on PrEP for serodifferent partners [32]. Peluso and colleagues developed a practical risk mitigation package during ATIs which encompass PrEP referral or provision paired with counseling for ATI participants and partners and HIV/ATI disclosure aids. However, this package was developed in the San Francisco context, where ATI trial participants tend to be older White/Caucasian men who have easier PrEP access [12]. Adaptation of risk mitigation approaches is needed in locales and populations where PrEP is not as well-known and readily available or acceptable [16].

To optimize choice, HIV serodifferent partners in our study stressed the importance of providing other HIV prevention options to ATI trial participants and their partners. This finding is consistent with a recent empirical ethics study aimed at increasing engagement of racial, ethnic, sex, and gender minority groups in ATI research [16]. While some participants in our study highlighted that abstinence and celibacy would constitute theoretical options, they felt these approaches would not be practical in the long-term, particularly with extended ATIs. They similarly were circumspect about condom use as a prevention tool, noting that over-reliance on condom use messages may be off-putting in communities where condomless sex has become the norm. HIV serodifferent partners provided alternative options to penetrative sex to maintain intimacy, and expressed other needs such as good communication, social support, and knowledge of how other couples were successfully managing and (re)-orienting their lives around ATIs.

For the most part, partners without HIV would want to be involved in ATI trial designs. HIV serodifferent partners highlighted the sense of togetherness in life, health, and research. These findings challenge the current paradigm in HIV cure clinical trial design where the partner with HIV is the only person implicated in research. The ethical conundrum of sex partners has been discussed in the literature [9, 11, 33]. Eyal advanced a ‘low-hanging fruit’ approach to risk mitigation for ATI non-participants, recommending prevention measures implemented across the arc of clinical research (e.g., recruitment, consent, ATI, and ART re-initiation) [10]. Similarly, bioethicist Dawson stressed the need for a relational approach to ATIs and professional ethical standards of nonmaleficence [11]. Dyads represent a crucial unit of analysis and intervention for basic HIV prevention efforts [34] and may similarly be important to consider in HIV cure research efforts. Dyadic approaches [35–38] – where members work together to achieve the shared goal of preventing HIV [34] – have great relevance for designing acceptable risk mitigation packages in ATI trials. Dyadic approaches can be tailored towards different types of sexual partnerships, ranging from one-time to enduring (e.g., long-term partnerships) [34].

There are a number of limitations to our study. This was a qualitative study in a small and non-randomly selected sample of diverse HIV serodifferent couples in the U.S., and thus the findings are exploratory and should be replicated in larger samples and with other types of couples. Due to the small sample size, we did not explore differences by geographic regions, and it is also possible that we did not achieve saturation in themes [29]. Additionally, the data were affected by the dynamic interaction of the couples during the interviews. In some interviews, the partner with HIV dominated the conversation, notwithstanding the interviewer’s attempts to actively engage the partner without HIV. Moreover, HIV serodifferent couples were interviewed together as a pair, leading to convergence of ideas; had the partners been interviewed separately, there may have been more divergence in perceptions. Future studies might interview both partners separately to triangulate the data obtained with interviews conducted with the couple as a unit. Due to time constraints, we did not capture length of time on ART, length or type of relationships, and we did not distinguish the specific nature of the couples’ relationship. Finally, all partners were in a known HIV serodifferent relationship since this was a study inclusion criterion. It is possible that partners who have not disclosed HIV status or are unaware of their HIV status may feel differently towards U = U and ATIs.

Table 3 provides a summary of findings and preliminary considerations for planning ATI trials with diverse HIV serodifferent partners.

**Table 3:** Summary of findings and preliminary considerations for planning ATI trials with racial, ethnic, sex and gender diverse HIV serodifferent partners

• It will be important to give PWH decision tools to make informed decisions around emerging HIV control options – including possible risks, benefits, and trade-offs. More emphasis should be dedicated to understanding unmet needs for PWH and their partners in the search towards an HIV cure. • There are mixed understandings of U = U in the community, even among HIV serodifferent partners. Planning ATI trials should occur concurrently with increased community engagement around treatment as prevention messages. Research teams should support PWH disclose loss of HIV undetectable status to their partners. • In the community, ATIs may be perceived as contradicting ART adherence messages and as a breach of U = U. The scientific rationale for ATIs used in HIV cure trials should be carefully communicated in lay terms. ATIs may cause worries and discomforts around passing or acquiring HIV, particularly among HIV serodifferent partners (and also secondary partners). • ATIs have several implications for both participants and partners – including the need for frequent viral load measurements, robust partner protection measures, good communication, and disclosure plans for both primary and secondary partners. Institutional review boards (IRBs) may consider asking ATI research teams to include risk mitigation plans as part of operations manuals. • Our study showed strong support for PrEP provision (and/or referral) during ATIs. ATI trials should be implemented jointly with efforts to increase PrEP awareness and access in the community. Research teams should emphasize the element of choice (e.g., daily PrEP, on-demand PrEP), and increase HIV prevention options available during ATI trials. • If possible, research teams should offer (peer) counseling and social support for both partners. Robust strength-based interventions emphasizing resilience should also be developed to help couples through ATIs. • For the most part, partners without HIV would like to be engaged in the ATI research process (even though they would not be considered ATI trial participants). Partners’ wishes should be respected with regards to their involvement in the research process. Dyads may represent critical units of analysis and interventions in the context of ATI trials.	

## Conclusions

To capture the voices of those who may benefit the most from an HIV cure, who are among the most highly affected by HIV due to intersectional identities and socio-contextual circumstances, and to acknowledge the relational aspects of trials involving ATIs, we spoke with a sample of HIV serodifferent partners spanning a wide range of racial, ethnic, sex and gender minority groups. The feasibility and long-term success of HIV cure research among highly affected racial, ethnic, sex, and gender diverse populations hinges on the needs of couples and their shared concerns for each other’s health. Future HIV research cure studies must integrate perspectives from biomedical and social-behavioral sciences (i.e., qualitative and mixed methods research) to effectively consider the complex dyadic environment and the ethical considerations to protect research participants and their sex partners from unanticipated harm due to their trial participation. Our study takes the first step in this direction; however, future studies should continue to explore these issues among other types of partnerships (i.e., non-monogamous, casual partnerships, polyamorous), cultures (i.e., racial and ethnic groups not included in the current study population (Asian, Native American/Indigenous, bi-racial), and socio-cultural settings (i.e., foreign born and mixed generational partners). Informing trials with diverse perspectives will make the eventual scale up of HIV cure strategies more acceptable among communities who need them the most.

## Supplementary Information

**Additional file 1: Supplementary Table 1.** Supplementary Quotes – In-Depth Interviews with Racial, Ethnic, Sex and Gender Diverse HIV Serodifferent Couples (United States, 2020).

## Data Availability

All relevant data related to this study have been included in the body of the manuscript and in Supplementary Table 1.
