# Supplementary material for: “It comes altogether as one:” perceptions of analytical treatment interruptions and partner protections among racial, ethnic, sex and gender diverse HIV serodifferent couples in the United States
Source: BMC Public Health. 2022 Jul 9;22:1317. doi: 10.1186/s12889-022-13528-8 (PMC9270765; doi:10.1186/s12889-022-13528-8)
Supplement: Supplementary file 1 — Additional file 1: Supplementary Table 1. Supplementary Quotes – In-Depth Interviews with Racial, Ethnic, Sex and Gender Diverse HIV Serodifferent Couples (United States, 2020). [file 12889_2022_13528_MOESM1_ESM.docx]

**Supplementary Table 1: Supplementary Quotes – In-Depth Interviews with**

**Racial, Ethnic, Sex and Gender Diverse HIV Serodifferent Couples (United States, 2020)**

| **Themes** | **Sub-Themes** | **Participants** | **Exemplar Quotes** |
| --- | --- | --- | --- |
| **Experiences with Current HIV Medications and Perceived Improvements in Antiretroviral Regimens** | | | |
| **Doing well on current HIV medications** | Minimal side effects | 01-Partner with HIV | So I’m goin’ stay with what I’m on, because I’m doing fine with it. No signs, no symptoms. I’m good to go. So and it’s a one day—it’s a one pill regimen. I’ve been on a 32 pills a day. So one pill a day is absolutely great for me. |
|  |  | 02-Partner with HIV | I'm taking a pill that I think is the smallest and it has less toxins than the one I was taking before |
| **Hesitancy around switching to newer ART regimens** | Association with weight gain | 01-Partner with HIV | But this next, the newest medication I heard is causing people to gain weight, and I’ve been there and I’ve done that, and [now] I’m doing well. |
| **Mixed feelings about LA-ART** | Would try LA-ART | 01-Partner with HIV | But they done switched us and switched us and switched us, and now it’s like this is the one. This is the one before the cure. |
|  |  | 03-Partner with HIV | Oh, that [long-acting ART] would be awesome. That would definitely be awesome. I wouldn’t mind that. I definitely wouldn’t mind that. |
|  |  | 05-Partner with HIV | Yeah, I'll take that. Better than taking it every day, I would take the shot. |
|  |  | 07-Partner with HIV | So then the pill wouldn’t be as much complicated to take it or not to take it as if it would be like—you know, versus injection or whatever. And so, that means your research will lead us to that point. That's why maybe it's good to do this kind of research, I think. |
|  | Would require more information | 03-Partner without HIV | I would be open to it. I would definitely want to know more information about it and probably ask questions of how that way have been working for most patients so far. And has there been any side effects or any concerns that patients that might have brought up. |
| **Understanding of Undetectable = Untransmittable (U = U)** | | | |
| **Mixed understanding of U = U** | Clear explanation of U = U | 06-Partner without HIV | Undetectable equals…untransmittable… My understanding, basically that if someone has undetectable viral load, that it is next to impossible for them to transmit the virus to another person. |
| **Perceptions of Analytical Treatment Interruptions (ATIs)** | | | |
| **Discomfort with ATIs** | Discomfort with partner with HIVs being off ART | 03-Partner without HIV | I just think that I would be uncomfortable with my partner like putting him to stop taking his medicine just for research. I guess if we were to participate in it I would just need more information. |
|  | Falling on one’s knees | 04-Partner with HIV | Oh, they're trying to find out how long if you stay off your medicine before you fall to your knees… Okay, they'd have to have a hospital bed right there, a doctor right there. They're going to have to have an intravenous thing right there, bring me back to life immediately… So that they can give me the real hot good shot, so if you about to die. |
|  | Partner would become widower | 05-Partner with HIV | I have to take my medicine. I wouldn't, like… if I did that study without taking the medicine, I think I'd probably get sick. When I don't take it, I start kind of shaking… If I don't take the medicine… my husband would be a widow[er]. |
| **Worries around Transmitting and Acquiring HIV** | | | |
| **Worries about transmitting HIV** | | 01-Partner with HIV | Don’t want to put nobody’s life in jeopardy |
|  |  | 04-Partner with HIV | Right. I wouldn't want to pass it to nobody… I don't want to pass no sickness to nobody. |
|  |  | 10-Partner with HIV | Yes. I would. I would worry because maybe I'd have a viral load again, and I wouldn't want to risk that with my partner… Even though [name of partner]'s on PrEP and he's safe, I still feel like it's up to me to maintain my own balance of having no viral load. So then I'm non-transmittable… Well, it would make me fearful. I mean, it goes back to that where I wouldn't want to risk becoming detectable again, because of infecting [name of partner]. So I would be hesitant to be in a study, unless I saw data that supported it very strongly and [name of partner] remained on PrEP, which he's on PrEP. Then I would consider it. |
| **Worries about acquiring HIV** | | 04-Partner without HIV | [W]hat I'm saying if there's a chance of you passing the shit, I don't want to take that chance. That's all I'm saying |
| **Partner Protection Measures** | | | |
| **PrEP** | Support for PrEP | 03-Partner with HIV | I actually believe just in wearing protection and shielding yourself or we heard about PREP, about the Truvada. He could use that, you know what I mean— |
|  |  | 03-Partner without HIV | [T]hey should be provided with all of the best suggestions as far as, what’s that called, Truvada PREP, and then also with using condoms and, you know, how all the of the things that that might be risky in the situation. They should be aware of anything that could possibly happen. |
|  |  | 07-Partner without HIV | PrEP is like protection, I think. Yeah, it's protection to avoid getting infected with HIV… I never used PrEP, but it's part of the research and you know, something good for you to be safe. |
|  |  | 09-Partner without HIV | Well, if for whatever reason—even if someone were practicing safe sex—if there was some possibility of transmission to someone else, then it would be better if they were taking PrEP. |
|  |  | 10-Partner with HIV | As part of the study, yes. I think that's a great idea. If you're taking the HIV positive partner and he's going off his meds to be on another medication, perhaps a cure, then to protect the partner by making sure that the partner is on PrEP. |
|  | PrEP hesitancy | 04 – Dyadic exchange | Truvada? Oh yeah, Truvada, yeah. – 04-Partner without HIV  …You don't want to take that crap. – 04-Partner with HIV  “Step up, PrEP up”, yeah. Uh-huh, I seen the commercial all the time. – 04-Partner without HIV  You don't want to do that... I don't want him to take that. – 04-Partner with HIV  …Yeah, I would consider it if it wouldn’t—yeah, if it's not gonna do anything to me to where I'll be cool, yeah. I wouldn't mind, "Step up, PrEP up"… Because I like the little commercial. – 04-Partner without HIV  Eat some rat poison. –04-Partner with HIV |
|  |  | 10-Partner without HIV | And I spoke with my doctor not even two days ago, and I said, "I'm kind of considering getting off PrEP," because I've read of some of the side effects of one of the components, the [commercial name]. |
| **Condoms** | Support for condoms | 03-Partner without HIV | I’d say condom use. |
|  |  | 04 – Dyadic exchange | But you've got to use rubbers, though… Rubbers. – 04-Partner with HIV  …Condoms all the time. – 04-Partner without HIV |
| **Monogamy** | Not stepping outside of the relationship | 03 – Dyadic exchange | Actually, I was saying basically that we don’t really step outside of the relationship. So the thing I was saying that another thing that we stay safe or to keep each other safe is that we don’t... – 03-Partner with HIV  By being monogamous. – 03-Partner without HIV  It’s no surprise, we stay monogamous. And being monogamous we have an understanding with that, you know what I mean? If we were to step out we would give each other knowledge of it. We would have knowledge of it. – 03-Partner with HIV |
| **Alternative to penetrative vaginal or anal sex** | Lack of penetrative sex | 03-Partner with HIV | We also don’t always penetrate. |
| **Taking oneself out of U = U pool** |  |  |  |
| **Counseling and social support** | Counseling | 10-Partner with HIV | Counseling, I think. |
|  | Social support | 01-Partner with HIV | I attend this group, sort of workshop that this HIV organization provides for people who are positive or negative. I think these workshops are very good for us, for people who are positive because we get educated, and then once you get educated, you can pass that information to other people. I think those workshops about talking about how to prevent HIV if you have it, what's the best way to have sex so you don't get HIV, these workshops are a good source for people who are concerned about HIV. |
|  |  | 09-Partner without HIV | Well, I think it would be good to have someone they could talk to, if any concerns come up during the trial. |
|  |  | 05-Partner without HIV | A support group to help the partner with [inaudible] your partner, with just going through [inaudible] somebody to stay there for support group to talk about what's going on. |
|  | Learning from other couples undergoing ATIs | 03-Partner without HIV | I will probably be interested in it while we’re – if we decide to do the study. And then also I would just want the research team to provide us with enough knowledge and we would also want to know how it probably have affected other clients, without knowing any personal information about them, but just a random overall opinion on how it has worked for other people participating in the study. |
| **Partner Consultation and Involvement in ATI Trials** | | | |
| Partner without HIV involved in ATI trial | Could provide support | 07 – Dyadic exchange | Well, in the case of my partner, we are together and it's important for me to always have him part of whatever I do. – 07-Partner with HIV  …Yes, because I know every detail. And I will know more what's going on and give support. –07-Partner without HIV |
